# Clinical efficacy and safety of porcine fibrin sealant in video-assisted thoracoscopic surgery lobectomy for lung cancer

**DOI:** 10.3389/fsurg.2026.1839723

**Published:** 2026-06-01

**Authors:** Teng Jia, Ming-hui Yue, Shu-jing Yin, Tian-yu She, Xiao-gang Zhao, Xiao-jie Gu

**Affiliations:** 1 First Clinical Medical College, Shandong University of Traditional Chinese Medicine, Jinan, Shandong, China; 2 Department of Thoracic Surgery, Binzhou Medical University Hospital, Binzhou, Shandong, China; 3 Department of Thoracic Surgery, The Second Qilu Hospital of Shandong University, Jinan, Shandong, China; 4 Department of Ultrasound, Binzhou Medical University Hospital, Binzhou, Shandong, China

**Keywords:** lung cancer, pleural effusion, porcine fibrin sealant, pulmonary air leak, VATS

## Abstract

**Objective:**

Lung cancer remains the leading cause of cancer-related mortality worldwide. Although minimally invasive surgery continues to advance, postoperative complications (such as pleural effusion and pulmonary air leaks) remain significant challenges, prolonging the duration of chest tube placement and increasing patient burden. This study aimed to systematically evaluate the safety and clinical efficacy of porcine fibrin sealant (PFS, a biological tissue adhesive), in VATS lobectomy for lung cancer.

**Methods:**

We conducted a single-center, retrospective observational cohort study including 345 lung cancer patients who underwent VATS lobectomy plus systematic lymph node dissection between 2017 and 2023. The patients were divided into a control group (*n* = 142) and a PFS group (*n* = 203) receiving intraoperative PFS application. The primary endpoints were total postoperative chest drainage volume and the duration of chest tube placement. Secondary endpoints included the incidence of pulmonary air leaks, severely prolonged drainage (>5 days), and other complications. PSM and OW models were employed to adjust for the baseline.

**Results:**

The unadjusted and PSM cohorts demonstrated the statistical benefits of PFS across multiple aspects. After rigorous adjustment using OW, the difference in total drainage volume was attenuated (*P* = 0.089). Nevertheless, in the OW cohort, PFS still significantly shortened the duration of chest tube placement (*P* = 0.036) and effectively decreased the occurrence of PAL (0.0% vs. 5.0%, *P* = 0.019) and severely prolonged drainage (0.0% vs. 22.3%, *P* < 0.001). There were no significant differences in other complications, and no allergic reactions occurred, indicating its good biosafety.

**Conclusion:**

The results demonstrate that the intraoperative application of PFS during VATS lobectomy is safe and effective. It can significantly prevent postoperative pulmonary air leaks and reduce delayed chest tube removal.

## 1 Introduction

Lung cancer is the leading cause of cancer-related mortality worldwide (1). Anatomical lobectomy remains the standard treatment for early-stage primary lung cancer (2). Although the development of minimally invasive techniques has significantly reduced surgical trauma, postoperative complications remain a major clinical challenge (3). Among these, pleural effusion caused by surgical surface exudation and pulmonary air leaks are the most common. These complications directly lead to a prolonged duration of chest tube drainage, which in turn increases the risk of infection and the economic burden for patients (4, 5).

To reduce the incidence of postoperative pulmonary air leaks and shorten the time to chest tube removal, materials including allogeneic/autologous fibrin sealants and human serum albumin-polyethylene glycol (PEG) polymers have been developed for lung volume reduction surgery in patients with emphysema. Studies have also explored their application in segmentectomy (6). However, these methods still carry certain risks, such as cross-infection (7, 8), or involve cumbersome extraction processes and are difficult to store long-term.

Porcine Fibrin Sealant (PFS), as a novel biological sealant, features high biosafety, immediate availability, and ease of use. PFS is primarily composed of fibrinogen, coagulation factor XIII, and thrombin. In the presence of Ca2+ ions, it rapidly cross-links to form a stable three-dimensional fibrin network (9). This network exerts a dual synergistic effect: on the one hand, it acts as a physical barrier to seal alveolar micro-fistulas; on the other hand, it provides a biological scaffold for the migration and proliferation of fibroblasts and capillaries, accelerating the repair of surgical surface tissues. It has been shown to effectively reduce the incidence of cerebrospinal fluid leaks following dural repair. However, whether its application in lobectomy for lung cancer yields clinical benefits remains unclear, and some studies advise against its routine use (10, 11). In light of this, the present study retrospectively analyzed 345 patients who underwent lobectomy for lung cancer. By employing Propensity Score Matching (PSM) and Overlap Weighting (OW) models to further balance inter-group differences, we systematically evaluated the impact of intraoperative PFS application on total postoperative chest drainage volume, duration of chest tube drainage, incidence of pulmonary air leaks, and postoperative complications, thereby exploring its safety and efficacy.

## 2 Materials and methods

This single-center, observational, retrospective cohort study included 345 patients with lung cancer who presented to the Department of Thoracic Surgery at Binzhou Medical University Hospital and underwent video-assisted thoracoscopic surgery (VATS) for radical resection of lung cancer (lobectomy and systematic lymph node dissection) between 2017 and 2023. General patient characteristics, chest drainage volume, and postoperative complications were collected. All participants signed written informed consent in accordance with the Declaration of Helsinki (2013 revision).

The inclusion criteria were as follows: (1) age > 18 years; (2) anatomical lobectomy plus systematic lymph node dissection; (3) postoperative pathological diagnosis of primary lung cancer.

The exclusion criteria were: (1) received preoperative chemotherapy or radiotherapy; (2) extensive pleural adhesions found intraoperatively; (3) intraoperative blood transfusion; (4) intraoperative blood loss exceeding 400 mL; (5) postoperative albumin <30 g/L; (6) combined with wedge resection, additional lobectomy, or other intrathoracic surgeries; (7) pathological diagnosis of carcinoma *in situ*; (8) pulmonary air leak lasting more than 7 days; (9) unplanned reoperation; (10) lymphatic fistula (chylothorax); (11) severe postoperative complications or death. Tumor-node-metastasis (TNM) staging was performed according to the International Association for the Study of Lung Cancer (IASLC) staging system (9th edition).

All radical resections for lung cancer in this study were performed via VATS. Intraoperatively, the division of incomplete fissures and bronchial closure were performed using mechanical staplers combined with an electrocautery hook or ultrasonic scalpel. Pulmonary vessels were ligated using Hem-o-lok clips or mechanical staplers. All enrolled patients underwent VATS lobectomy and systematic lymph node dissection (for left-sided tumors, lymph nodes at stations 5, 6, 7, 9, 10, 11, and 12 were completely dissected; for right-sided tumors, lymph nodes at stations 2R, 4R, 7, 9, 10, 11, and 12 were completely dissected). In the PFS group only, PFS (Bioseal®, Guangzhou Bioseal Biotech Co., Ltd., 5.0 mL) was sprayed over the lymph node dissection areas and the bronchial and vascular stumps.

It is important to note that the use of PFS in patients was not randomized. This decision primarily depended on the operating surgeon's clinical preference and their real-time assessment of the surgical field. Specifically, the application of PFS was mainly based on the evaluation of the patient's risk for pulmonary air leaks, such as the presence of incomplete lung fissures or underlying emphysema. Given this non-randomized allocation and intraoperative decision-making process, inherent selection bias is inevitable. Therefore, the results of this study should be interpreted as demonstrating an association. Meanwhile, to rigorously control for these baseline confounders and mitigate selection bias, we further employed PSM and OW models.

Application and dosage of PFS: After being removed from the refrigerator, the PFS was warmed to room temperature to facilitate dissolution. Before spraying the PFS, the target area was cleaned with endoscopic gauze to ensure optimal adhesion of the sealant. The PFS was sprayed 1–2 cm above the surgical field using a disposable endoscopic catheter connected to the top of a double-barrel applicator. After 3–5 s, a translucent milky gel formed. The sealant was allowed to completely solidify before the re-expansion of the remaining lung, to prevent it from adhering to the lung surface and being dislodged from the target area.

Considering inadequate pleural drainage caused by postoperative pain and routine postoperative monitoring, and to minimize the risk of secondary thoracentesis, the timing of chest tube removal in this study was set to at least 3 days postoperatively, provided that patients met the following criteria: (1) satisfactory lung re-expansion on chest radiography; (2) no air bubbles in the water-seal chamber during coughing; (3) absence of bright red bloody or turbid pleural effusion; and (4) pleural fluid volume not exceeding 200 mL on the day of chest tube removal.

The primary endpoints were the total postoperative chest drainage volume and the duration of chest tube placement. Secondary endpoints included the incidences of severely prolonged drainage (defined as >5 days), prolonged air leak, pulmonary infection, and other overall postoperative complications.

### 2.1 Statistical analysis

Statistical analyses were performed using R software. Continuous variables are expressed as mean ± standard deviation (SD) or median with interquartile range (IQR) and were compared using the Student's *t*-test or Mann–Whitney U test, as appropriate. Categorical variables are presented as frequencies and percentages and were compared using the Pearson chi-square test or Fisher's exact test. To mitigate selection bias, we employed PSM and OW. The propensity score (PS) for receiving PFS treatment was estimated using a multivariable logistic regression model incorporating patient demographics, tumor characteristics, and relevant baseline covariates. For PSM, a 1:1 nearest-neighbor matching algorithm without replacement was utilized. To maximize the retention of the cohort sample size and achieve more precise covariate balance, we primarily relied on OW. In the OW scheme, patients in the PFS group were assigned a weight of (1-PS), while those in the control group were assigned a weight of PS. Covariate balance was evaluated using the standardized mean difference (SMD), with an SMD < 0.10 indicating good balance. Time-to-event outcomes were assessed using Kaplan–Meier survival curves and the log-rank test.

## 3 Results

### 3.1 Patient selection and baseline characteristics

**Figure 1:**
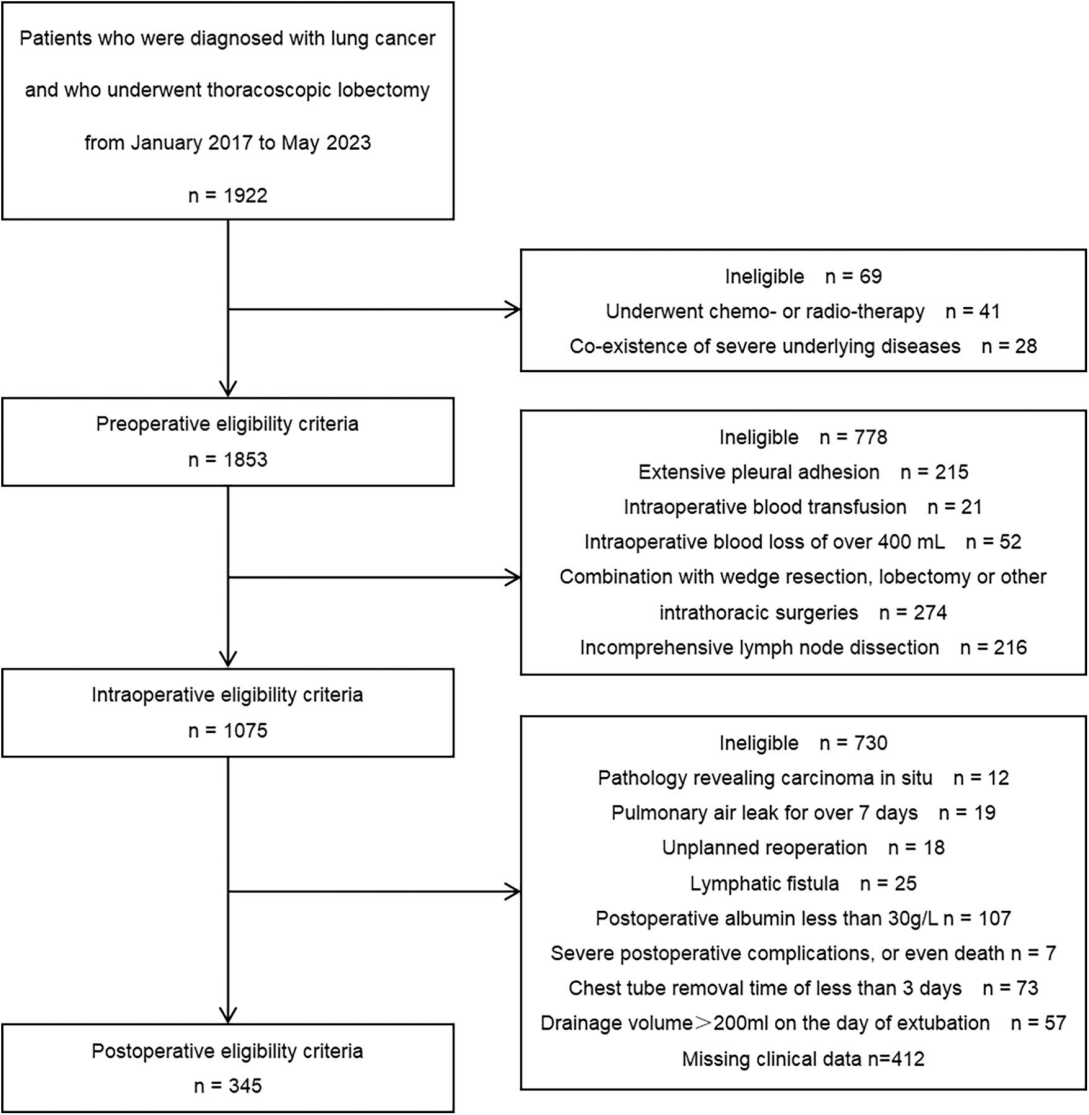
Flowchart of patient inclusion and exclusion criteria.

**Figure 2:**
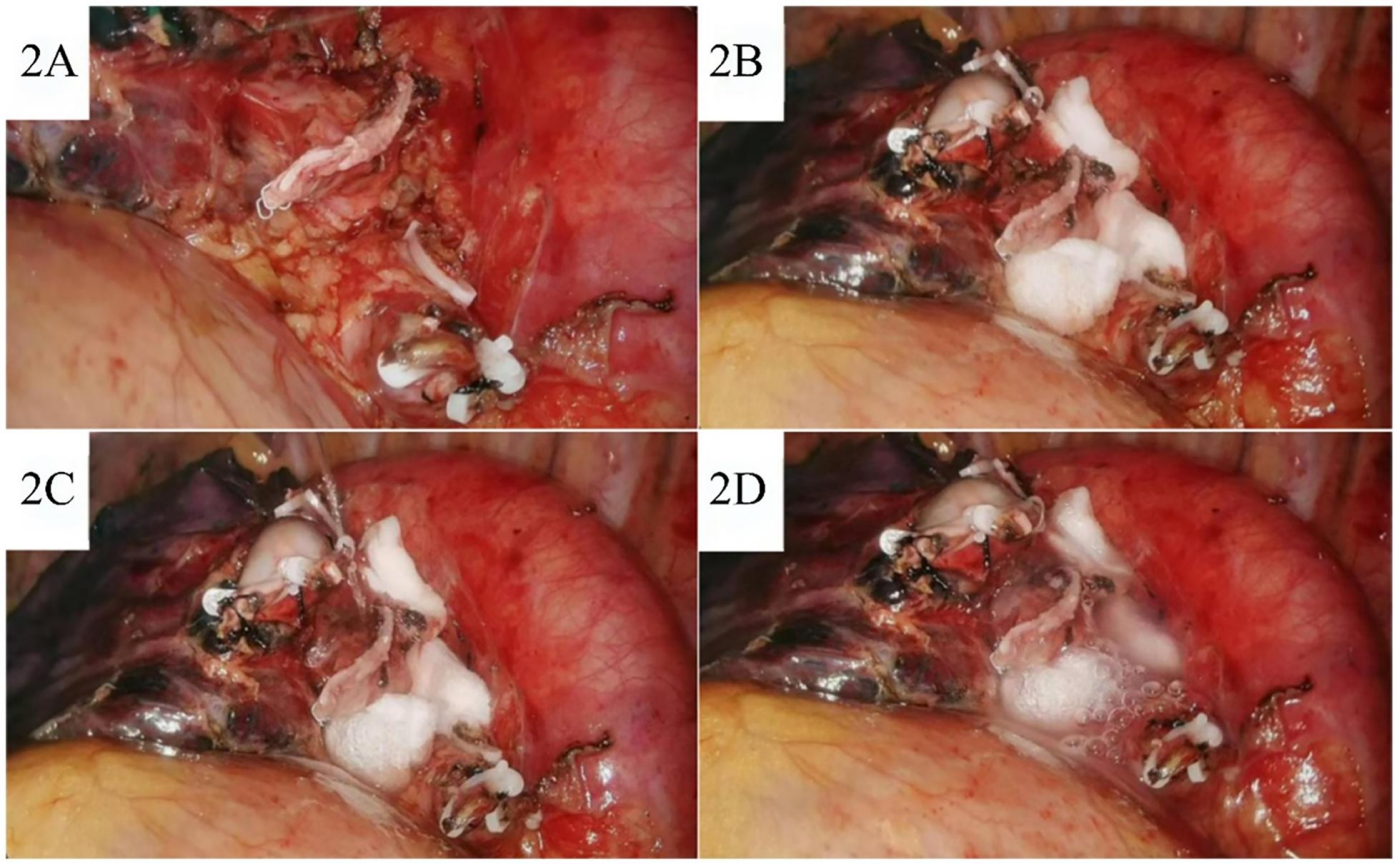
Condition of the surgical surface before and after PFS application. **(A)** Postoperative photograph of thoracoscopic lobectomy. **(B)** Hemostatic dressings were placed on the mediastinal wounds after lymph node dissection. **(C)** Porcine fibrin sealant being sprayed into the surgical areas using the disposable endoscope catheter. **(D)** The translucent milky gel was formed after spraying porcine fibrin sealant.

**Figure 3:**
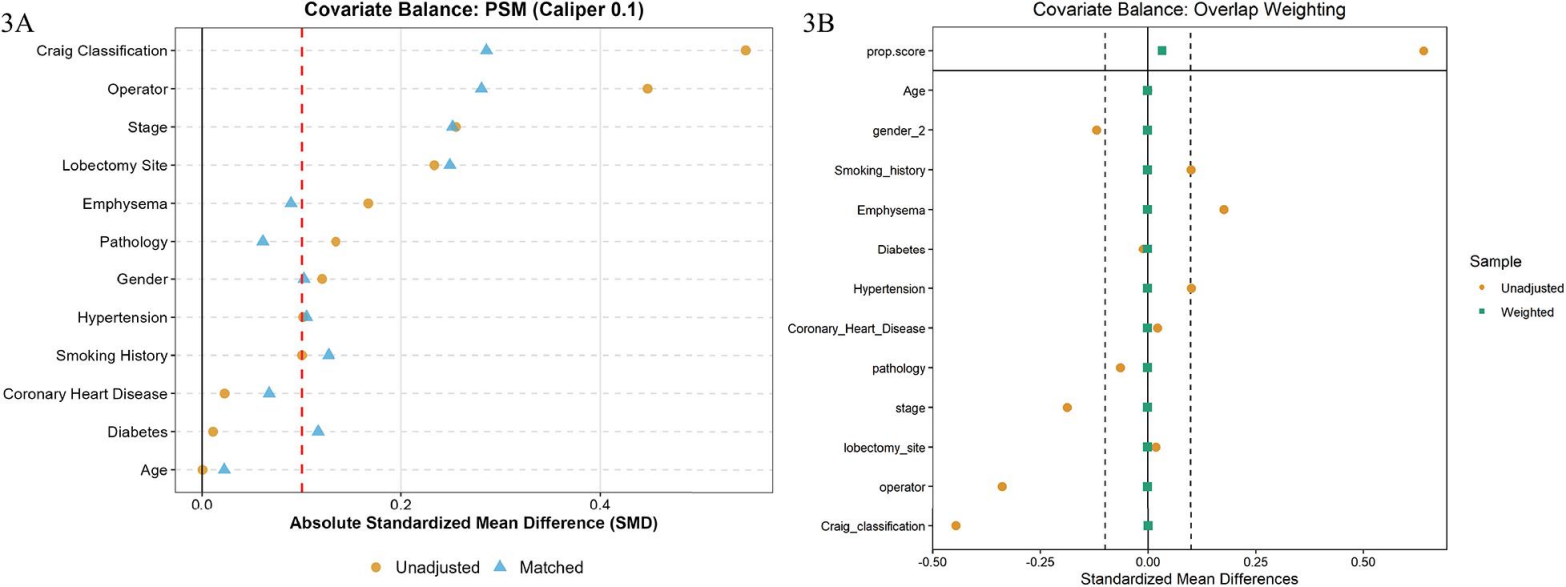
Comparison of covariate balance. **(A)** absolute SMD distribution before and after PSM. The red dashed line indicates the 0.10 threshold. **(B)** SMD distribution before and after OW. The black dashed lines delineate the ±0.1 threshold range.

Ultimately, a total of 345 eligible patients were included in the final analysis (Figure 1: Flowchart of patient inclusion and exclusion criteria). Among them, 142 patients were in the control group and 203 patients were in the PFS group (Figure 2: Condition of the surgical surface before and after PFS application). Statistical differences were observed between the two groups regarding the Operator and Craig classification (Table 1: Baseline characteristics of patients in the unadjusted, PSM, and OW cohorts). To further enhance comparability between the groups, we performed propensity score matching (PSM) analysis; however, variables including Stage, Lobectomy site, Operator, and Craig classification remained inadequately balanced (SMD > 0.1). Therefore, overlap weighting (OW) was applied for further analysis in this study. Following OW adjustment, a perfect balance was achieved across all baseline characteristics between the two groups, with all SMDs < 0.1 (Table 1), indicating a high degree of comparability in the distribution of patient characteristics (Figure 3).

**Table 1:** Baseline characteristics of patients in the unadjusted, PSM, and OW cohorts.

Characteristic	Group	Total Participants				Propensity Score Matching Group				Overlap Weighting Group			
		Control	PFS	*P* value	SMD	Control	PFS	*P* value	SMD	Control	PFS	P_Value	SMD
Age		58.91 (8.40)	58.91 (7.63)	0.997	<0.001	58.72 (7.98)	58.90 (7.95)	0.864	0.022	59.12 (8.12)	59.12 (7.59)	1	<0.001
Gender	Male	58 (40.8)	95 (46.8)	0.325	0.12	48 (40.3)	54 (45.4)	0.513	0.102	40.40%	40.40%	1	<0.001
	Female	84 (59.2)	108 (53.2)			71 (59.7)	65 (54.6)			59.60%	59.60%		
Smoking_history	No	101 (71.1)	135 (66.5)	0.429	0.1	85 (71.4)	78 (65.5)	0.402	0.127	71.20%	71.20%	1	<0.001
	Yes	41 (28.9)	68 (33.5)			34 (28.6)	41 (34.5)			28.80%	28.80%		
Emphysema	No	131 (92.3)	177 (87.2)	0.187	0.167	110 (92.4)	107 (89.9)	0.648	0.089	91.60%	91.60%	1	<0.001
	Yes	11 (7.7)	26 (12.8)			9 (7.6)	12 (10.1)			8.40%	8.40%		
Diabetes	No	124 (87.3)	178 (87.7)	1	0.011	106 (89.1)	110 (92.4)	0.502	0.116	86.80%	86.80%	1	<0.001
	Yes	18 (12.7)	25 (12.3)			13 (10.9)	9 (7.6)			13.20%	13.20%		
Hypertension	No	95 (66.9)	126 (62.1)	0.42	0.101	78 (65.5)	72 (60.5)	0.502	0.105	65.10%	65.10%	1	<0.001
	Yes	47 (33.1)	77 (37.9)			41 (34.5)	47 (39.5)			34.90%	34.90%		
Coronary_Heart_Disease	No	133 (93.7)	189 (93.1)	1	0.022	112 (94.1)	110 (92.4)	0.796	0.067	93.10%	93.10%	1	<0.001
	Yes	9 (6.3)	14 (6.9)			7 (5.9)	9 (7.6)			6.90%	6.90%		
Pathology	Adenocarcinoma	129 (90.8)	190 (93.6)	0.454	0.134	110 (92.4)	108 (90.8)	0.896	0.061	92.90%	92.90%	1	<0.001
	Squamous cell	9 (6.3)	7 (3.4)			5 (4.2)	6 (5.0)			3.90%	3.90%		
	Neuroendocrine	4 (2.8)	6 (3.0)			4 (3.4)	5 (4.2)			3.20%	3.20%		
Stage	IA	89 (62.7)	143 (70.4)	0.327	0.255	82 (68.9)	84 (70.6)	0.446	0.252	67.40%	67.40%	1	<0.001
	IB	26 (18.3)	31 (15.3)			20 (16.8)	19 (16.0)			16.90%	16.90%		
	IIA	5 (3.5)	6 (3.0)			4 (3.4)	2 (1.7)			3.70%	3.70%		
	IIB	9 (6.3)	15 (7.4)			5 (4.2)	10 (8.4)			6.70%	6.70%		
	IIIA	12 (8.5)	8 (3.9)			8 (6.7)	4 (3.4)			5.20%	5.20%		
	IIIB	1 (0.7)	0 (0.0)			0 (0.0)	0 (0.0)			0.00%	0.00%		
Lobectomy_site	Left upper	31 (21.8)	44 (21.7)	0.368	0.234	27 (22.7)	22 (18.5)	0.457	0.249	22.60%	22.60%	1	<0.001
	Left lower	26 (18.3)	32 (15.8)			24 (20.2)	23 (19.3)			19.40%	19.40%		
	Right upper	50 (35.2)	71 (35.0)			41 (34.5)	44 (37.0)			34.00%	34.00%		
	Right midder	8 (5.6)	24 (11.8)			6 (5.0)	13 (10.9)			6.70%	6.70%		
	Right lower	27 (19.0)	32 (15.8)			21 (17.6)	17 (14.3)			17.30%	17.30%		
Operator	Junior	17 (12.0)	32 (15.8)	<0.001	0.448	16 (13.4)	9 (7.6)	0.1	0.281	15.10%	15.10%	1	<0.001
	Intermediate	25 (17.6)	70 (34.5)			23 (19.3)	35 (29.4)			22.60%	22.60%		
	Senior	100 (70.4)	101 (49.8)			80 (67.2)	75 (63.0)			62.30%	62.30%		
Craig_classification	Grade 1	8 (5.6)	24 (11.8)	<0.001	0.546	7 (5.9)	9 (7.6)	0.19	0.286	8.30%	8.30%	1	<0.001
	Grade 2	51 (35.9)	86 (42.4)			51 (42.9)	43 (36.1)			41.20%	41.20%		
	Grade 3	60 (42.3)	89 (43.8)			51 (42.9)	63 (52.9)			46.10%	46.10%		
	Grade 4	23 (16.2)	4 (2.0)			10 (8.4)	4 (3.4)			4.40%	4.40%		

### 3.2 Impact of PFS on pleural drainage

**Figure 4:**
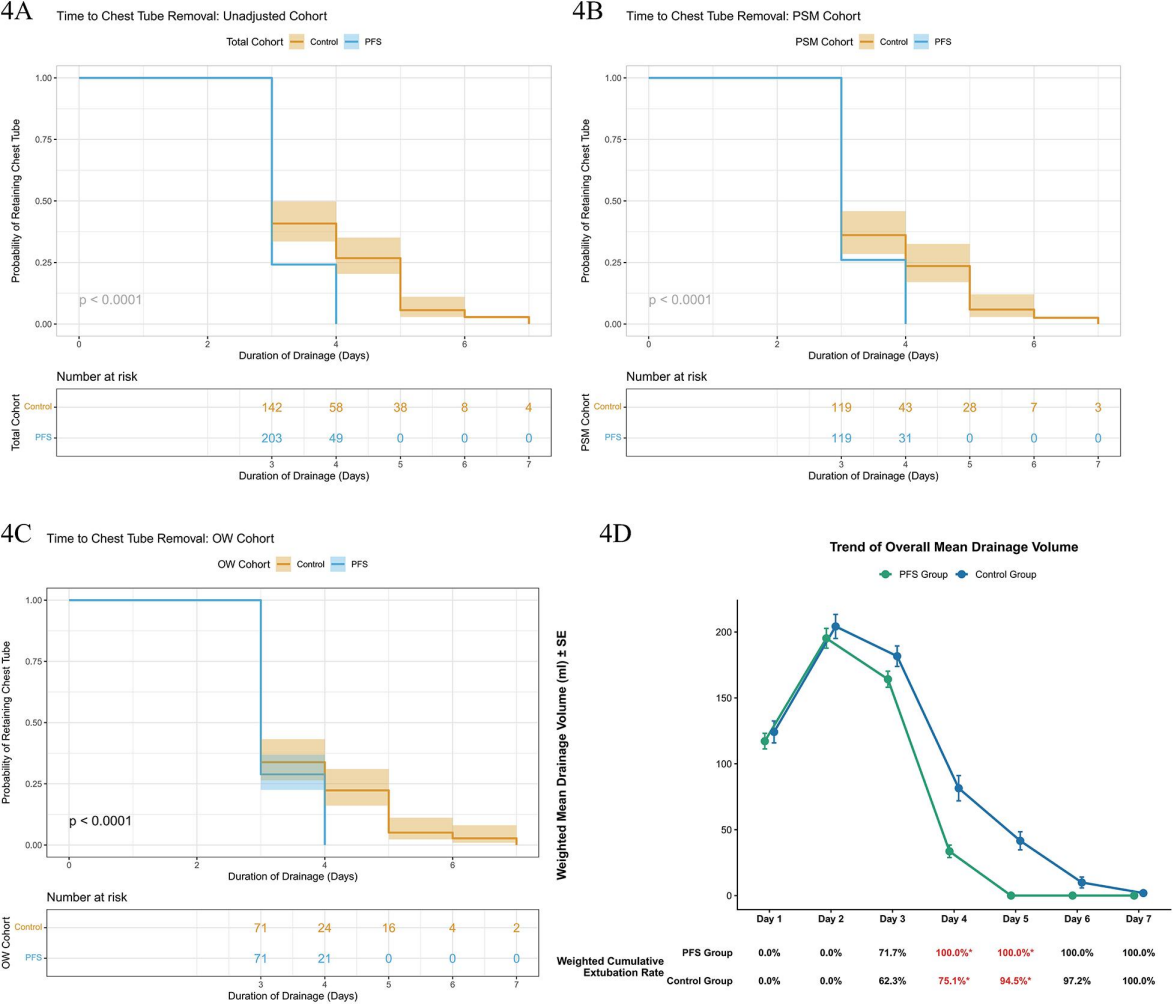
Impact of PFS on chest tube removal and drainage. **(A–C)** Kaplan–Meier plots comparing the probability of retaining a chest tube over time in the Control and PFS groups across the Unadjusted, PSM, and OW cohorts, respectively (Log-rank *P* < 0.0001). **(D)** Trend plot in the OW cohort illustrating dynamic changes in weighted mean daily drainage volume (mL ± SE), showing a more rapid decrease in the PFS group after Day 3.

In the initial unadjusted overall cohort, compared with the control group, the application of PFS was associated with a reduction in total drainage volume (median 460.00 mL vs. 547.50 mL, *P* < 0.001) and a shortened duration of chest tube placement (*P* < 0.001). We further confirmed this trend in the Kaplan–Meier curves (Log-rank *P* < 0.001, Figure 4A). Furthermore, there were no recorded cases of severely prolonged chest tube placement of ≥5 days in the PFS group (0.0% vs. 26.8%, *P* < 0.001).

After performing PSM, PFS continued to provide clinical benefits. The total drainage volume in the PFS group was significantly reduced (median 460.00 mL vs. 515.00 mL, *P* = 0.034), while the duration of chest tube placement continued to be shortened (*P* = 0.008), which was consistent with the results of the Kaplan–Meier analysis (Log-rank *P* < 0.001, Figure 4B). The preventive effect on severely prolonged chest tube placement of ≥5 days was also maintained (0.0% vs. 23.5%, *P* < 0.001).

Finally, after rigorous adjustment using OW to completely eliminate baseline differences, the difference in total drainage volume was attenuated (median 480.00 mL vs. 500.58 mL, *P* = 0.089). Nevertheless, the duration of chest tube placement remained significantly shorter in patients receiving PFS treatment (*P* = 0.036), and a statistical difference was also observed in the duration of chest tube placement between the PFS and Control groups in the Kaplan–Meier analysis (Log-rank *P* < 0.001, Figure 4C). Most importantly, in the fully adjusted OW cohort, the preventive effect of PFS on severely prolonged chest tube placement remained significant (0.0% vs. 22.3%, *P* < 0.001) (Figure 4D illustrates the changes in patient drainage volume).

### 3.3 Postoperative complications

We systematically evaluated the impact of PFS on postoperative complications in the unadjusted, PSM, and OW cohorts (Table 2). Across all cohorts, the incidences of pulmonary infection, atrial fibrillation, pulmonary edema, and pulmonary embolism showed no statistically significant differences between the PFS and control groups (*P* > 0.05). This indicates that the intraoperative application of PFS does not increase the risk of these adverse events.

**Table 2:** Incidence of postoperative complications.

Postoperative outcomes	Group	Total Participants			Propensity Score Matching Group			Overlap Weighting Group		
		Control	PFS	*P* value	Control	PFS	*P* value	Control	PFS	*P* value
Total_drainage		547.50 (380.00, 880.00)	460.00 (330.00, 620.00)	<0.001	515.00 (352.50, 875.00)	460.00 (350.00, 620.00)	0.034	500.58 (350.00, 860.00)	480.00 (355.00, 639.31)	0.089
Duration_of_drainage		3.00 (3.00, 5.00)	3.00 (3.00, 3.00)	<0.001	3.00 (3.00, 4.00)	3.00 (3.00, 4.00)	0.008	3.00 (3.00, 4.00)	3.00 (3.00, 4.00)	0.036
Prolonged_drainage ≥ 5d	No	104 (73.2)	203 (100.0)	<0.001	91 (76.5)	119 (100.0)	<0.001	77.70%	100.00%	<0.001
	Yes	38 (26.8)	0 (0.0)		28 (23.5)	0 (0.0)		22.30%	0.00%	
Complications	No	127 (89.4)	192 (94.6)	0.115	107 (89.9)	112 (94.1)	0.339	89.10%	95.60%	0.03
	Yes	15 (10.6)	11 (5.4)		12 (10.1)	7 (5.9)		10.90%	4.40%	
Prolonged_air_leak	No	136 (95.8)	203 (100.0)	0.011	11 (9.2)	7 (5.9)	0.071	95.00%	100.00%	0.019
	Yes	6 (4.2)	0 (0.0)		114 (95.8)	119 (100.0)		5.00%	0.00%	
Pulmonary_infection	No	135 (95.1)	196 (96.6)	0.683	5 (4.2)	0 (0.0)	1	95.20%	97.30%	0.309
	Yes	7 (4.9)	7 (3.4)		113 (95.0)	113 (95.0)		4.80%	2.70%	
Atrial_Fibrillation	No	140 (98.6)	201 (99.0)	1	6 (5.0)	6 (5.0)	1	98.80%	99.10%	0.825
	Yes	2 (1.4)	2 (1.0)		118 (99.2)	119 (100.0)		1.20%	0.90%	
Pulmonary_edema	No	142 (100.0)	202 (99.5)	1	1 (0.8)	0 (0.0)	NA	100.00%	99.80%	0.32
	Yes	0 (0.0)	1 (0.5)		119 (100.0)	119 (100.0)		0.00%	0.20%	
Pulmonary_embolism	No	142 (100.0)	202 (99.5)	1	0 (0.0)	1 (0.8)	1	100.00%	99.40%	0.317
	Yes	0 (0.0)	1 (0.5)		119 (100.0)	118 (99.2)		0.00%	0.60%	

Furthermore, PFS demonstrated a clear clinical benefit in reducing the incidence of postoperative pulmonary air leaks. In the initial unadjusted cohort, the incidence of pulmonary air leaks was significantly lower in the PFS group (0.0%) compared to the control group (4.2%, *P* = 0.011). This effect was maintained in the PSM cohort (4.7% in the control group, *P* = 0.070) and also showed a statistically significant difference in the OW cohort (0.0% in the PFS group vs. 5.0% in the control group, *P* = 0.019). These findings suggest that PFS possesses excellent biosafety while offering advantages in reducing the risk of pulmonary air leaks following lobectomy.

## 4 Discussion

Enhanced Recovery After Surgery (ERAS) is currently a crucial strategy in the perioperative management of thoracic surgery, which requires us to attenuate postoperative stress responses and reduce the incidence of pulmonary complications (12). An increase in the duration of chest tube placement significantly prolongs the patient's pain cycle and increases the likelihood of infection, which is detrimental to rapid recovery. Previous studies have shown that fibrin sealants have a certain effect on improving chest tube duration. To our knowledge, this is the largest retrospective study to date evaluating the use of PFS after lobectomy for lung cancer. In this study, after introducing PSM and OW models to balance baseline confounders, PFS still shortened the duration of chest tube placement to some extent. However, the criteria for chest tube removal in this study were relatively strict (placement for at least > 3 days and <200 mL on the third day); this phenomenon might be masked under more lenient ERAS removal criteria (daily drainage volume < 400 mL), especially given that PFS did not significantly alter the total pleural drainage volume of patients in this study.

Postoperative pulmonary air leak is one of the main bottlenecks hindering early chest tube removal in thoracic surgery patients (4, 13). During anatomical lobectomy, the destruction of capillaries is inevitable, and alveolar micro-fistulas may occur. The role of PFS in improving postoperative pulmonary air leaks cannot be ignored. The application value of autologous fibrin sealants in lung volume reduction surgery for patients with severe emphysema has been confirmed (14). In this study, after adequately balancing baseline bias via OW, our findings demonstrated a effective reduction in the incidence of pulmonary air leaks following PFS application, which is consistent with the performance of autologous fibrin sealants. Based on this, although PFS did not significantly reduce the pleural drainage volume, its performance regarding pulmonary air leaks can improve the safety of chest tube removal, especially in the context of Enhanced Recovery After Surgery (ERAS), relatively aggressive chest tube removal strategies are generally encouraged. However, concerns regarding secondary interventions caused by postoperative pulmonary air leaks may, to some extent, hinder the broader implementation of ERAS pathways. The clinical utility of PFS may largely depend on institutional chest tube management strategies. In centers adopting more aggressive ERAS protocols, the reduction in postoperative pulmonary air leak risk associated with PFS may further translate into shorter chest tube duration and reduced length of hospital stay. Therefore, future prospective studies within standardized ERAS perioperative pathways are still warranted to further evaluate the clinical value of PFS.

As a foreign body compared to autologous fibrin sealants, the animal-derived nature of PFS brings attention to allergic reactions. Encouragingly, no obvious allergic reactions were observed among patients in the PFS group in this study. Furthermore, the application of PFS did not increase the incidence of other cardiopulmonary complications, indicating its excellent biosafety.

This study has certain limitations. First, it is a single-center, retrospective observational study. Although we employed statistical methods such as propensity score matching (PSM) and overlap weighting (OW) to maximally balance baseline differences between groups, it is inevitable that some unmeasured confounders (e.g., the surgeon's learning curve) might still influence the results. Second, we applied extensive exclusion criteria in our study design. While this rigorous selection process enhanced the internal validity of our analysis, it inevitably introduced a certain degree of selection bias, which may limit the external validity of our findings and their generalizability in real-world clinical practice. Third, due to the retrospective nature of this study, a more objective method (such as the Maccearini classification) was not employed to evaluate the severity of pulmonary air leaks, and the severity of emphysema was not effectively recorded. Consequently, further stratified analysis could not be performed. Fourth, as mentioned previously, our center adopted a relatively strict standardized pathway for chest tube removal (mandating chest tube placement for at least 3 days); this intervention model might have masked the potential true benefits of PFS in promoting early chest tube removal (e.g., on postoperative day 1 or 2). Fifth, this study primarily focused on perioperative and short-term postoperative clinical outcomes, lacking follow-up data on long-term effects on patients. For instance, whether PFS exerts a sustained impact on the long-term recovery of patient pulmonary function remains unknown; furthermore, for lung cancer patients undergoing radical resection, whether this foreign material might appear on imaging and affect subsequent treatment decisions requires investigation. Therefore, large-sample, multicenter, prospective randomized controlled trials (RCTs) with long-term follow-up designs are still needed in the future to further comprehensively evaluate the overall clinical value and long-term safety of PFS.

## 5 Conclusion

The application of PFS in video-assisted thoracoscopic surgery (VATS) lobectomy can reduce delayed chest tube removal in patients to a certain extent. Additionally, PFS can reduce the incidence of postoperative pulmonary air leaks. As an important adjunctive measure in thoracic surgery, PFS can influence early chest tube removal to a certain degree while enhancing the safety of removal, perfectly aligning with the clinical pathway of ERAS.

## Data Availability

The datasets generated and/or analyzed during the current study are not publicly available due to patient privacy. Requests to access the datasets should be directed to author TJ, jiateng517@yeah.net.
